# Antithrombotic strategies for preventing graft failure in coronary artery bypass graft

**DOI:** 10.1007/s11239-023-02940-5

**Published:** 2024-03-16

**Authors:** Maria Sara Mauro, Simone Finocchiaro, Dario Calderone, Carla Rochira, Federica Agnello, Lorenzo Scalia, Davide Capodanno

**Affiliations:** Division of Cardiology, Azienda Ospedaliero Universitaria Policlinico “G. Rodolico-San Marco”, University of Catania, Via Santa Sofia, 78, Catania, Italy

**Keywords:** Coronary artery bypass grafting, Antithrombotic therapy, Graft failure, Coronary artery disease.

## Abstract

Coronary artery bypass graft (CABG) procedures face challenges related to graft failure, driven by factors such as acute thrombosis, neointimal hyperplasia, and atherosclerotic plaque formation. Despite extensive efforts over four decades, the optimal antithrombotic strategy to prevent graft occlusion while minimizing bleeding risks remains uncertain, relying heavily on expert opinions rather than definitive guidelines. To address this uncertainty, we conducted a review of randomized clinical trials and meta-analyses of antithrombotic therapy for patients with CABG. These studies examined various antithrombotic regimens in CABG such as single antiplatelet therapy (aspirin or P2Y_12_ inhibitors), dual antiplatelet therapy, and anticoagulation therapy. We evaluated outcomes including the patency of grafts, major adverse cardiovascular events, and bleeding complications and also explored future perspectives to enhance long-term outcomes for CABG patients. Early studies established aspirin as a key component of antithrombotic pharmacotherapy after CABG. Subsequent randomized controlled trials focused on adding a P2Y_12_ inhibitor (such as clopidogrel, ticagrelor, or prasugrel) to aspirin, yielding mixed results. This article aims to inform clinical decision-making and guide the selection of antithrombotic strategies after CABG.

**Supplementary Information:**

The online version contains supplementary material available at 10.1007/s11239-023-02940-5.

## Introduction

Coronary artery bypass grafting (CABG) is widely accepted as a treatment option for patients with left main or multivessel coronary artery disease [1]. However, after CABG, patients remain at risk of coronary events due to the progression of their underlying atherosclerosis or the failure of the arterial conduits or saphenous vein grafts (SVG) used during the procedure [2]. Despite efforts to prioritize total arterial revascularization, SVG continue to be the predominant choice, and the incidence of SVG failure remains high, with reported rates ranging from 3 to 12% [3].

Early failure of arterial or saphenous grafts is typically attributed to acute thrombosis, while long-term failure results from thrombosis, the development of atheromatic plaques, or neointimal hyperplasia [4]. The occlusion of grafts due to thrombosis is influenced by various factors, including alterations in local blood hemodynamics and changes to the vessel wall [4, 5]. These processes trigger increased platelet activation, underscoring the essential role of antithrombotic therapy in any strategy aimed at preserving graft patency and preventing ischemic complications [6]. However, the optimal approach to antithrombotic management is more uncertain after a CABG procedure compared to percutaneous coronary intervention, where the evidence is more robust. Current guidelines offer suggestions on the choice and duration of antiplatelet therapy after CABG, but the evidence supporting these recommendations is limited and primarily based on expert opinions. Nevertheless, recent studies have emerged that offer new insights in this space [7, 8].

This article aims to provide an update on the use of antithrombotic therapy for the purpose of preventing graft failure after a CABG procedure.

## Mechanisms of graft failure

**Fig. 1:**
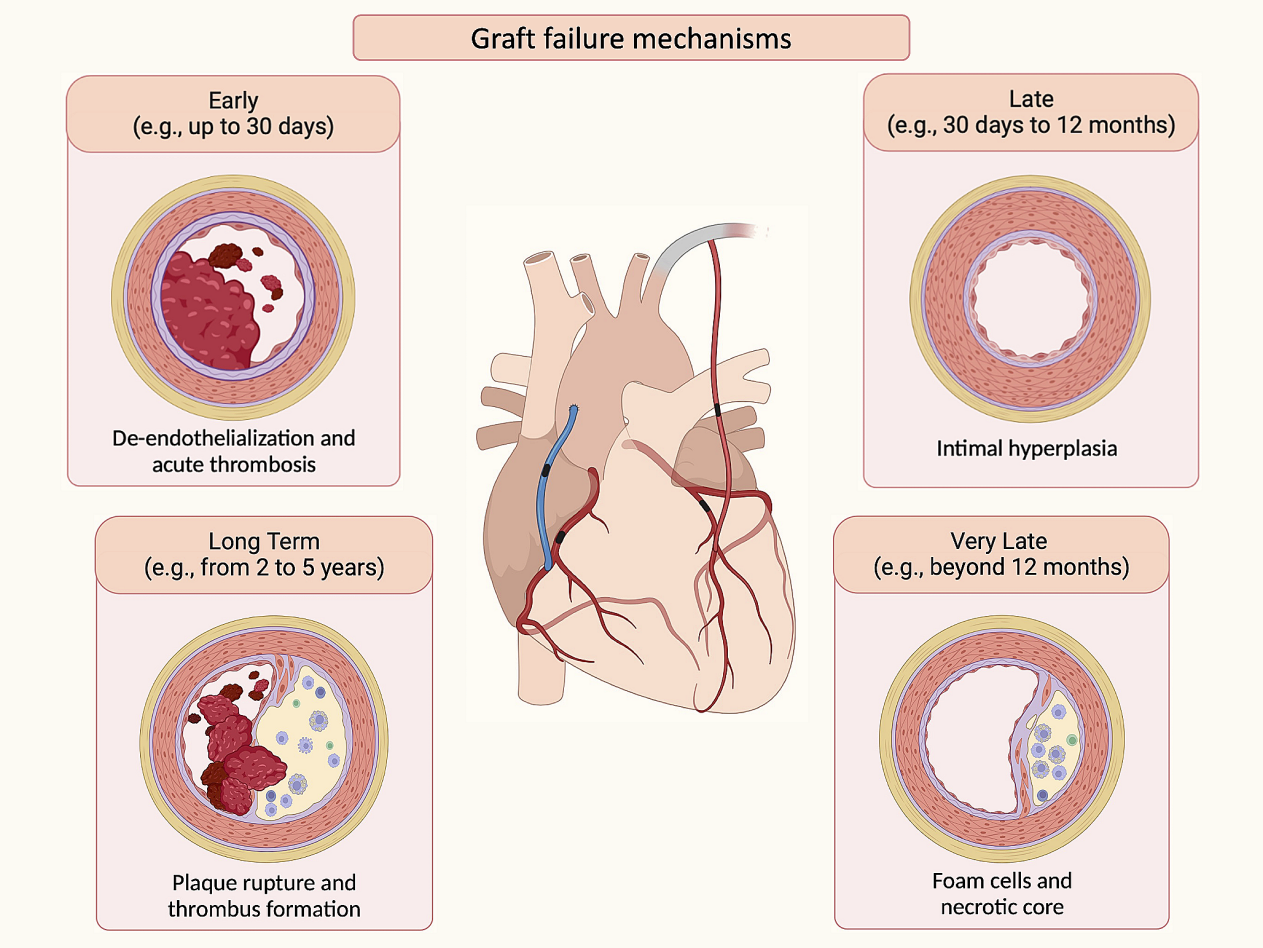
**Mechanisms of Graft Failure in CABG**. Graft failure in CABG is a complex process involving various pathophysiological mechanisms. Early, late, very late, and long-term graft failures can be attributed to distinct factors and processes

Graft failure results from multiple underlying pathophysiological processes (Fig. 1). Early graft failure (i.e., within hours to less than a month) is generally attributed to acute thrombosis. During the harvesting process, mechanical forces and ischemia-reperfusion injury result in damage to the endothelial cells and smooth muscle cells [9, 10]. The resulting reduced levels of prostacyclin and nitric oxide activate leukocytes and platelets, which mediate thrombus formation by adhering to the extracellular matrix and producing thrombogenic factors such as platelet-derived growth factor, transforming growth factor β, fibrinogen, fibronectin, and von Willebrand factor [11].

Between one month and one-year post-CABG, the leading cause of SVG failure is intimal hyperplasia. These cases are often characterized by concentric and diffuse atherosclerosis of the graft, lacking a fibrous cap and is more susceptible to rupture due to rapid progression [12]. Conversely, graft failure beyond 12 months is more frequently characterized by the accumulation of foam cells and the growth of a necrotic core with cholesterol deposits. This event typically occurs two to five years after the procedure, starting with intermediate lesions. The expansion of the necrotic core due to intraplaque hemorrhage from neoangiogenic vessels leads to plaque rupture and thrombus formation. Arterial grafts are known to have a more resistant atheroma plaque capsule compared to SVGs, making the latter more susceptible to plaque rupture and thrombosis [12].

Systemic risk factors, such as diabetes mellitus and aging, play a crucial role in determining the success of CABG by promoting a pro-atherogenic phenotype. A lower individual response to antithrombotic therapy after CABG in patients with high platelet reactivity can also increase the risk of early or late graft failure [13].

## Evidence review

**Fig. 2:**
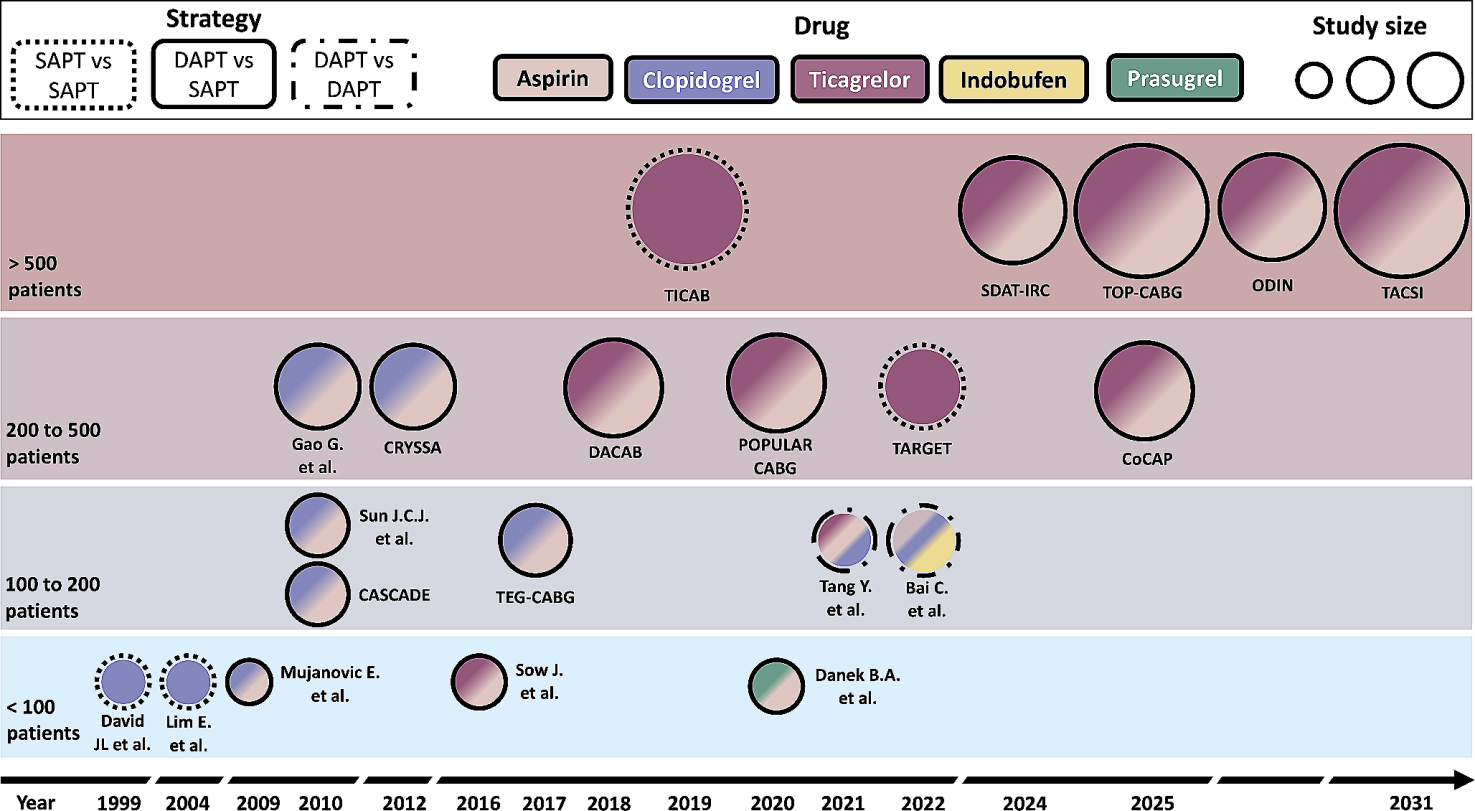
**Randomized clinical trials of antiplatelet therapy after CABG.** The figure presents a visual representation of randomized clinical trials investigating antiplatelet therapy following CABG

Figure 2 shows the timeline of key randomized clinical trials of antiplatelet therapy after CABG.

### Single antiplatelet therapy

Early placebo-controlled studies published in the eighties tested warfarin, indobufen, aspirin at different doses and the combination of high-dose aspirin and dipyridamole, with mixed results [14–23]. In the largest of these studies (*n* = 555) all the aspirin-based regimens improved graft patency at 60 days from surgery [19]. These results were consistent in a smaller trial (*n* = 231) where an aspirin dose of 324 mg daily, given within one hour after CABG, resulted in a significant reduction in SVG occlusion at 1 week that was sustained at one year [18]. Following these studies, aspirin became a pillar of secondary prevention in this setting [24, 25]. However, the response to aspirin can be highly variable among CABG patients [26, 27]. Therefore, alternative antiplatelet agents, including the P2Y_12_ receptor inhibitors clopidogrel and ticagrelor, have been investigated (Table 1).

**Table 1:** Randomized trials comparing monotherapy regimens after CABG

RCTs, Year	Sample size	Interventional arm	Control arm	Follow-up	Primary efficacy endpoint	Primary safety endpoint
David JL et al., 1999	62	Clopidogrel (C) 50 mg/die or 75 mg/die or 100 mg/die	Ticlopidine (T) 250 mg/bid	28 days	Ex-vivo platelet aggregation: Day 9: inhibition in the T group but not in the C groups (*p* < 0.01); Day 28: equally significant inhibition in the T, C100 and C75 groups (*p* < 0.001) and at a less extent in the C50 group (*p* < 0.01)	BT was significantly prolonged versus baseline in the T, C100 and C75 (*p* < 0.001). The prolongation was significant but at a less extent in the C50 group (*p* < 0.05)
Lim E. et al., 2004	54	Clopidogrel (C) 75 mg/die	ASA (A) 100 mg or 325 mg	5 days	Mean percentage aggregations with collagen: 56% for A and 99% for C; mean difference between the two arms was 42% (95% CI, 27 − 56%) in favor of A	NA
TiCAB, 2019	1,859	Ticagrelor (T) 90 mg/bid	ASA (A) 100 mg/die	12 months	Composite of cardiovascular death, MI, repeat revascularization, and stroke: HR 1.19; 95% CI 0.87 to 1.62; *p* = 0.28	BARC ≥ 4 for periprocedural and hospital stay-related bleedings and BARC ≥ 3 for post-discharge bleedings: HR 1.17; 95% CI 0.71 to 1.92; *p* = 0.53
TARGET, 2022	250	Ticagrelor (T) 90 mg/bid	ASA (A) 81 mg/bid	12 months	SVG occlusion: 13.2% vs. 17.4%; *p* = 0.30	Freedom from major adverse cardiovascular events; *p* = 0.60

**Abbreviations**: ASA, aspirin; BARC, Bleeding Academic Research Consortium; BID, bis in die; BT, bleeding time; CI confidence interval; HR, hazard ratio; MI, myocardial infarction; NA, not available; SVG, saphenous vein graft

#### Clopidogrel

Clopidogrel irreversibly inhibits the platelet response through mechanisms mediated by adenosine diphosphate. A subgroup analysis of patients undergoing cardiac surgery in the CAPRIE trial (including but not limited to patients undergoing CABG) suggested that clopidogrel might be better than aspirin in the context of long-term management [28, 29]. On the other hand, several pharmacodynamic studies failed to demonstrate a relevant early effect of clopidogrel in comparison with other single antiplatelet agents. For example, a study of 62 patients compared different doses of clopidogrel (i.e., 50, 75, or 100 mg) with ticlopidine 250 mg given twice daily, and found that all the three doses effectively inhibited platelet activity ex-vivo and prolonged bleeding time at day 28, but did not significantly reduce platelet aggregation at day 9 [30]. Another small study of 54 patients compared the effect of two doses of aspirin (100 mg or 325 mg) with clopidogrel 75 mg, and found no significant benefit of clopidogrel on platelet aggregation during the first five postoperative days [31]. As such, clopidogrel monotherapy is not currently recommended over aspirin to prevent the risk of early graft failure.

#### Ticagrelor

Ticagrelor reversibly binds to the platelet P2Y_12_ receptor and provides strong and rapid inhibition of adenosine diphosphate -induced platelet aggregation. The TiCAB trial compared ticagrelor monotherapy (90 mg, twice daily) with aspirin monotherapy (100 mg/day) during the first year after arterial and/or SVG implantation [32]. The trial was discontinued prematurely due to withdrawal of funding support from the sponsor, at a time when 1859 out of 3850 planned patients were randomized. No significant differences were observed between ticagrelor and aspirin in terms of major adverse cardiac events (MACE) at 12 months after CABG. However, these results should be interpreted with caution as the study was underpowered. The TARGET trial was another relatively small study (*n* = 250) comparing ticagrelor and aspirin after CABG [33]. The primary outcome was occlusion of SVG as determined by computed tomography coronary angiography at 12 months. There was no significant difference between the two groups [33], which was also confirmed at two years in 142 patients undergoing another computed tomography coronary angiography assessment [34]. In aggregate, similarly to clopidogrel, there is no evidence to recommend ticagrelor as single antiplatelet therapy after CABG.

### Dual antiplatelet therapy

Randomized clinical trials of DAPT for the prevention of graft occlusion are summarized below and in Table 2.

**Table 2:** Randomized clinical trials of dual versus single antiplatelet strategies after CABG

RCTs, Year	Sample size	N grafts	Graft type	Interventional arm	Control arm	Follow-up	Graft assessment method	Graft occlusion (any grafts, %)	SVGs occlusion (%)
Gao G. et al., 2010	249	704	SVGs (68%), LIMA, RA	ASA 100 mg plus clopidogrel 75 mg	ASA 100 mg	3 months	CTA	6.5 vs. 10.3 (*p* = 0.07)	8.4 vs. 14.3 (*p* = 0.04)
Mujanovic E. et al., 2009	20	56	SVGs (64%), LIMA	ASA 100 mg plus clopidogrel 75 mg	ASA 100 mg	3 months	Coronary angiography	6.9 vs. 29.6 (*p* = 0.04)	10.5 vs. 47.1 (*p* = 0.02)
CRYSSA, 2012	300	960	SVGs (57%), LIMA, RIMA, RA	ASA 100 mg plus clopidogrel 75 mg	ASA 100 mg	12 months	CTA	4.84 vs. 8.35 (*p* = 0.03)	7.4 vs. 13.1 (*p* = 0.04)
Sun J.C.J. et al., 2010	100	395	SVGs (58%), LIMA, RIMA, RA	ASA 81 mg plus clopidogrel 75 mg	ASA 81 mg plus placebo	1 months	CTA	5.0 vs. 7.1 (*p* = 0.43)	6.5 vs. 6.8 (*p* = 0.92)
CASCADE, 2010	113	NA	SVGs and arterial grafts	ASA 162 mg plus clopidogrel 75 mg	ASA 162 mg plus placebo	12 months	Coronary angiography with IVUS	4.8 vs. 4.5 (*p* = 0 0.90)	5.7 vs. 6.8 (*p* = 0.69)
TEG-CABG, 2017	165	355	SVGs (58%), LIMA, RIMA, RA	ASA 75 mg plus clopidogrel 75 mg	ASA 75 mg	3 months	CTA	25.7 vs. 22.4 (*p* = 0.84)*	11.9 vs. 6.7 (*p* = 0.29)
TAP-CABG, 2016	70	207	SVGs (48%) LIMA, RA	ASA 81 mg plus ticagrelor 90 mg bid	ASA 81 mg plus placebo	3 months	CTA	10.3 vs. 18.3 (*p* = 0.11)	10.0 vs. 22.0 (*p* = 0.12)
DACAB, 2018	500	1891	SVGs (77%), LIMA, RA	Ticagrelor 90 mg bid or ASA 100 mg plus ticagrelor 90 mg bid	ASA 100 mg	12 months	CTA or coronary angiography	NA	17.2 vs. 23.5 (*p* = 0.10) 11.3 vs. 23.5 (*p* < 0.001)
POPULAR-CABG, 2020	499	1847	SVGs (58%), LIMA, RIMA, RA	ASA (100 or 80 mg) plus ticagrelor 90 mg bid	ASA (100 or 80 mg) plus placebo	12 months	CTA	NA	9.6 vs. 10.1 (*p* = 0.64)
Danek B.A. et al., 2020	84	NA	SVGs	ASA 100 mg plus prasugrel 10 mg/die	ASA 100 mg plus placebo	12 months	Coronary angiography with OCT, IVUS and NIRS	NA	*p* = 0.06
Tang Y. et al., 2021	147	480	SVGs (70%), LIMA	ASA 100 mg plus ticagrelor 90 mg bid	ASA 100 mg plus clopidogrel 75 mg	12 months	CTA	6.7 vs. 7.5 (*p* = 0.73)	9.0 vs. 10.1 (*p* = 0.75)
Bai C. et al., 2022	152	540	SVGs (75%) and LIMA	Indobufen 100 mg bid plus clopidogrel 75 mg	ASA 100 mg plus clopidogrel 75 mg	12 months	CTA or coronary angiography	4.9 vs. 7.4 (*p* = 0.22)	5.5 vs. 8.7 (*p* = 0.21)

**Abbreviations**: ASA, aspirin; BID, bis in die; CTA, computed tomography angiography; IVUS, intravascular ultrasound; LIMA, left internal mammary artery; NA, not available; NIRS, near-infrared spectroscopy; OCT, optical coherence tomography; SVG, saphenous vein graft; RA, radial artery; RIMA, right internal mammary artery. *Rate of significant stenosis (> 50%) or occlusions

#### DAPT with aspirin and clopidogrel

Several studies compared the combination of clopidogrel and aspirin versus aspirin monotherapy, with small study samples and mixed findings. Four studies have reported improved graft patency [35–38]. The largest of these studies was the CRYSSA trial (*n* = 300), which showed a significantly lower risk of graft occlusion at 12 months [37]. Conversely, four other studies presented negative findings [39–42].

No trials of DAPT with clopidogrel and aspirin exist that were powered for hard clinical endpoints. Data from observational studies and post-hoc analyses of randomized trials designed for other purposes are available, but likely fraught by confounding bias. If anything, these studies were consistent in showing no benefit on mortality with clopidogrel in addition to aspirin. In the 2,072 patients with acute coronary syndromes (ACS) who received CABG in the CURE trial, DAPT reduced the risk of cardiovascular death, myocardial infarction, or stroke by 11%, but increased the risk of hemorrhagic complications by 30% [43]. Conversely, in a large retrospective registry from China (*n* = 18,069), CABG patients with DAPT had a lower incidence of all-cause death, stroke, myocardial infarction, or repeat revascularization at six months and had no differences in bleeding events [44]. In a sub-analysis of the ROOBY trial, DAPT increased early death (i.e., within 30 days) and did not improve the risk of death at long-term [45]. Additionally, in a study including aspirin-resistant patients, DAPT did not result in reduced death at six months [46]. These results were consistent with a post hoc analysis of the FREEDOM trial, where no differences in death, adverse ischemic events and bleeding was observed at five years among patients with type II diabetes mellitus undergoing CABG [47].

#### DAPT with aspirin and ticagrelor

The TAP-CABG trial (*n* = 70), which was terminated prematurely because of slow recruitment, evaluated the incidence of arterial and venous graft patency at 3 months after DAPT with ticagrelor and aspirin versus aspirin alone. The primary endpoint was slightly improved with DAPT (*p* = 0.044), but the difference was not significant in analyses stratified by individual grafts [48]. The larger DACAB trial randomized 500 patients to DAPT, ticagrelor alone or aspirin alone [49]. DAPT significantly improved the rate of SVG patency at 12 months compared to aspirin (risk difference, 12.2; 95% confidence interval [CI], 5.2–19.2%; *p* < 0.001). This effect was consistent in a post hoc analysis restricted to patients with ACS, who represented 67% of the entire population. The incidence of ischemic and bleeding events was low, which precludes the interpretation of clinical endpoints. A 5-year follow-up extension study of DACAB, where more events will be accrued, is ongoing (NCT03987373).

At variance with DACAB, the POPULAR-CABG trial (*n* = 499) showed no significant difference in one-year SVG patency with aspirin and ticagrelor compared to aspirin alone [50]. The different results of DACAB and POPULAR-CABG have two contributing explanations. Firstly, in DACAB, a higher proportion of patients underwent CABG for ACS than in POPULAR-CABG (i.e., two thirds versus one third). ACS is known as the population that benefits the most from a ticagrelor-based DAPT. Secondly, the use of cardiopulmonary bypass was markedly lower in DACAB (25%) than in POPULAR-CABG (95%). The impact of this difference on graft patency is unclear. Two recent meta-analyses suggested that the antiplatelet regimens that include ticagrelor are associated with improved clinical outcomes and increased graft patency [51, 52]. However, the findings of these meta-analyses were mixed regarding the risk of clinically important bleeding. In view of the conflicting results of the available studies, the efficacy of DAPT with ticagrelor and aspirin in improving the patency of SVGs remains undefined.

#### DAPT with aspirin and prasugrel

A recent study compared DAPT with prasugrel and aspirin versus aspirin alone [53], but was prematurely stopped due to slow enrolment after randomizing only 84 patients. The primary endpoint, the incidence of optical coherence tomography-detected SVG thrombus at 12 months, was observed in approximately one-third of the patients, without a significant difference between the two treatment groups. Additionally, there were no significant differences in angiographic SVG failure, the incidence of MACE, or severe bleeding. Two meta-analyses suggested that DAPT with prasugrel reduces the risk of SVG failure, mortality and MACE when compared with single antiplatelet therapy, albeit at the expense of an increased risk of major bleeding [54, 55].

#### Comparisons of DAPT strategies

Although the evidence in this area is not robust, some post-hoc analyses of studies comparing different DAPT strategies are informative. DAPT with aspirin and clopidogrel was compared to DAPT with aspirin and ticagrelor in the PLATO trial, which included 1,261 patients with ACS undergoing CABG [56]. The results in this subgroup showed a significant reduction in the composite outcomes of all-cause death, myocardial infarction, or stroke at 12 months with aspirin and ticagrelor, and similar rates of hemorrhagic events. Additionally, a pharmacodynamic study conducted in 140 patients undergoing CABG demonstrated that the onset of action was faster and the inhibition of platelet aggregation was higher with ticagrelor and aspirin than with clopidogrel and aspirin, with no difference in bleeding or MACE [57]. Another small trial (*n* = 147) reported similar rates of SVG patency at 1-year with ticagrelor-based and clopidogrel-based DAPT [58].

The only available data comparing DAPT with prasugrel and aspirin and DAPT with clopidogrel and aspirin comes from a subset analysis of the TRITON-TIMI 38 trial, which included 346 patients with ACS undergoing CABG [59]. Despite an increase in bleeding and surgical re-exploration, prasugrel-based DAPT was associated with a lower rate of death within 30 days after CABG. It is possible that the greater degree of platelet inhibition provided by prasugrel may have contributed to both the increased non-fatal bleeding and the reduced risk of fatal cardiac events and mortality. This evidence is mostly derived from sub-analyses of trials with non-stratified randomization, and therefore is not sufficient to draw definitive conclusions.

A recent Chinese trial (*n* = 152) compared DAPT with indobufen and clopidogrel to DAPT with aspirin and clopidogrel and found similar patency rates of SVG and arterial grafts at 12 months [60]. This trial also showed a similar rate of MACE between the two groups and a lower incidence of gastrointestinal adverse events in the indobufen group. Based on these findings, indobufen might be considered in DAPT combinations if aspirin is not an option.

### Anticoagulant therapy

Early studies investigated the effectiveness of various anticoagulants in preventing graft occlusion after CABG.

#### Vitamin K antagonists

In 1993, a meta-analysis of 17 trials concluded that warfarin significantly reduces the risk of graft occlusion compared to placebo, similar to aspirin [61]. No difference between vitamin K antagonists (VKA; i.e., acenocoumarol or phenprocoumon) and aspirin was demonstrated on SVG patency at one year in a trial of 948 patients [62].

In the landmark Post-CABG (Post-Coronary Artery Bypass Graft) trial, 1,351 patients on aspirin were randomized to low-dose warfarin (e.g., dual-pathway inhibition) or placebo [63]. While no significant effect was observed on progression of SVG disease, there were a 35% reduction in mortality (*p* = 0.008) and a 31% reduction of death or nonfatal myocardial infarction (*p* = 0.003) with warfarin and aspirin at 7.5 years [64]. The mechanism leading to such effects remained unexplained and play of chance cannot be ruled out. Indeed, only 11% of patients were on VKA during the extended follow-up.

#### Direct oral anticoagulant

More recently, there has been interest in evaluating a strategy of combining a direct oral anticoagulant (DOAC) with an antiplatelet agent. The rationale for this strategy is to reduce the degree of platelet activation throughout synergistic inhibition of thromboxane A_2_ production by aspirin and inhibition of thrombin and fibrin formation by the DOAC [65]. Due to lack of dedicated trials, whether this strategy is suitable for secondary prevention after CABG is unclear [66, 67].

In a prespecified substudy of the COMPASS trial, 1,448 patients were randomized within 4 to 14 days after CABG to rivaroxaban 2.5 mg twice daily plus aspirin 100 mg daily, rivaroxaban 5 mg twice daily, or aspirin 100 mg daily [68]. At an average of 1.13 years, compared to aspirin alone, rivaroxaban did not reduce the rate of both arterial and SVG failure either as a combination with aspirin or as monotherapy. Additionally, the two rivaroxaban-based strategies did not reduce the risk of a composite of cardiovascular death, stroke, or myocardial infarction and increased the risk of bleeding at 30 days after CABG. Notably, when compared to aspirin alone, the combination of rivaroxaban and aspirin did not increase the rate of graft patency in both patients treated with on-pump and off-pump techniques. Conversely, rivaroxaban monotherapy improved the rate of graft patency in patients undergoing off-pump CABG (odds ratio 0.37; 95% CI, 0.16 to 0.82; *p* = 0.01), but not in those undergoing on-pump CABG. Overall, these results do not support the use of rivaroxaban, either alone or in a dual-pathway inhibition regimen, after CABG. Further studies are warranted to corroborate the promise of rivaroxaban in patients undergoing off-pump CABG.

#### Parenteral anticoagulation

Another approach to prevent early graft failure is the use of parenteral anticoagulants such as fondaparinux. In the Fonda-CABG study, 99 CABG patients on aspirin therapy were randomized to fondaparinux 2.5 mg/daily or heparin in the early postoperative in-hospital period [69]. After discharge and up to 30 days, the fondaparinux group continued to receive fondaparinux, while the heparin group received placebo. Computed tomography angiography performed at 30 days demonstrated similar rates of graft occlusion and no statistically significant difference in death, stroke, myocardial infarction, bleeding events, or re-operation. Although it was not adequately powered for efficacy, the trial showed no benefit of extended fondaparinux therapy compared with heparin for the prevention of early graft failure.

## Guidelines

In the context of antithrombotic therapy for patients with CABG, the current guidelines on myocardial revascularization from the European Society of Cardiology (ESC) and the European Association for Cardio-Thoracic Surgery (EACTS) largely rely on the Focused Update on DAPT in Coronary Artery Disease published by the ESC in 2017 [8]. This document summarizes the findings of two meta-analyses comparing graft patency in patients receiving aspirin monotherapy versus DAPT with aspirin and clopidogrel [70, 71]. The majority of patients included in these meta-analyses had stable coronary artery disease, and both studies demonstrated a significant reduction in SVG occlusions with the use of DAPT. Nevertheless, given the low thrombotic risk after CABG in patients with stable coronary artery disease and the limited evidence, the guidelines do not generally recommend DAPT for preventing SVG in this setting [8].

In patients with ACS treated with DAPT and undergoing CABG, resumption of P2Y_12_ inhibitor therapy as soon as deemed safe after surgery and continuation up to 12 months is recommended by the ESC (class of recommendation I, level of evidence C) [8]. Additionally, the guidelines suggest that CABG patients at high ischemic risk and prior myocardial infarction, who have tolerated DAPT without experiencing bleeding complications, may be considered for treatment with DAPT for longer than 12 months and up to 36 months (class of recommendation IIb, level of evidence C) [72]. Conversely, in CABG patients with prior myocardial infarction who are at high risk of bleeding, discontinuation of P2Y_12_ inhibitor therapy after six months should be considered (class of recommendation IIa, level of evidence C) [8].

Finally, current guidelines do not support the routine use of VKAs to prevent graft occlusion after CABG, unless other indications for long-term anticoagulation coexist (e.g., atrial fibrillation, venous thromboembolism, mechanical prosthetic valves) [73, 74].

## Future directions

The current evidence on antithrombotic therapy after CABG is characterized by diverse and sometimes contradictory findings. Several ongoing trials are actively addressing the unanswered questions regarding the optimal pharmacotherapy for this specific patient population (Table 3).

**Table 3:** Ongoing randomized clinical trials of antithrombotic strategies after CABG

Trial name, NCT	Sample size	Population	Interventional arm	Control arm	Primary endpoint	Follow-up	Estimated study completion
TACSI, NCT03560310	2200	CABG in acute coronary syndromes	Ticagrelor plus aspirin	Aspirin	MACE	12 months	2031
CoCAP, NCT04783701	360	CABG in acute coronary syndromes	Ticagrelor plus aspirin	Aspirin	Graft patency	12–36 months	2025
SDAT-IRC, NCT03789916	800	Incomplete revascularization after CABG	Ticagrelor plus aspirin	Aspirin	Cardiovascular death	5 years	2024
TOP-CABG, NCT05380063	2300	CABG with SVG ≥ 1	Aspirin plus ticagrelor for 3 moths, followed by aspirin plus placebo for 9 months	Aspirin plus ticagrelor	SVGs occlusion, bleeding BARC ≥ 2	12 months	2025
ODIN (announced)	700	CABG in chronic coronary syndromes	Aspirin plus ticagrelor for 1 moth, followed by aspirin alone for 11 months	Aspirin	Not available	Not available	Not available

**Abbreviations**: BARC, Bleeding Academic Research Consortium; CABG, coronary artery bypass graft; MACE, major adverse cardiac events; SVG, saphenous vein graft

The ongoing TACSI (NCT03560310) trial is investigating whether DAPT with ticagrelor and aspirin reduces the risk of MACE at 12 months compared to aspirin alone in ACS patients undergoing CABG [75]. The CoCAP (NCT04783701) trial, an extension of TACSI, is assessing graft patency using computed tomography or coronary angiography at 12 to 32 months. The SDAT-IRC (NCT03789916) trial is examining the five-year efficacy of DAPT with ticagrelor and aspirin versus aspirin monotherapy in patients with incomplete revascularization. Additionally, as noted above, a follow-up extension of the DACAB trial (NCT03987373) will provide five-year outcomes of DAPT with ticagrelor and aspirin.

Two trials are focusing on short DAPT strategies. The TOP-CABG trial (NCT05380063) is comparing the non-inferiority of a three-month DAPT with ticagrelor and aspirin followed by aspirin monotherapy to standard DAPT in preventing SVG occlusion and reducing the risk of bleeding [76]. The ODIN trial (announced) will evaluate the efficacy of one month of DAPT with aspirin plus ticagrelor followed by 11 months of aspirin monotherapy compared to aspirin alone in patients undergoing CABG for chronic coronary syndromes. These trials aim to provide evidence supporting the safe reduction of antithrombotic therapy duration and intensity, minimizing bleeding complications without compromising graft patency.

## Conclusions

In this review, we explored the current evidence on antiplatelet and anticoagulant therapies for patients undergoing CABG. Early studies established aspirin as a key component of antithrombotic pharmacotherapy after CABG. Subsequent randomized controlled trials focused on adding a P2Y_12_ inhibitor (such as clopidogrel, ticagrelor, or prasugrel) to aspirin, with conflicting results. In most studies, DAPT demonstrated significant benefits in reducing SVG occlusion and improving graft patency, particularly in patients with ACS. However, this benefit was accompanied by an increased risk of bleeding. Current guidelines support the use of DAPT for 12 months in ACS patients, but not in those with stable coronary artery disease. The use of oral anticoagulants is limited to patients with other indications for long-term anticoagulation.

Overall, the optimal antithrombotic regimen for patients undergoing CABG remains a subject of debate. Considering the evolving surgical techniques that minimize endothelial injury and promote early graft healing, the exploration of short-term DAPT regimens, akin to interventional cardiology, offers a potential balance between graft patency and bleeding risk. However, larger randomized studies, including ongoing clinical trials, are needed to provide more definitive evidence and guidance regarding antithrombotic therapies in this patient population. These studies will contribute to shaping the optimal antithrombotic strategies for patients undergoing CABG.

### Electronic supplementary material

Below is the link to the electronic supplementary material.

Supplementary Material 1

## Data Availability

No new data were generated or collected specifically for this review.

## Abbreviations and acronyms

ACS: Acute coronary syndromes
CABG: Coronary artery bypass graft
DAPT: Dual antiplatelet therapy
DOAC: Direct oral anticoagulant
MACE: Major adverse cardiovascular events
MI: Myocardial infarction
SVG: Saphenous vein grafts

## References

[1] Doenst T (2019). PCI and CABG for treating stable coronary artery disease: JACC Review topic of the Week. J Am Coll Cardiol.

[2] Fitzgibbon GM (1996). Coronary bypass graft fate and patient outcome: angiographic follow-up of 5,065 grafts related to survival and reoperation in 1,388 patients during 25 years. J Am Coll Cardiol.

[3] Guida GA, Angelini GD (2022). Pathophysiology and mechanisms of Saphenous Vein Graft failure. Braz J Cardiovasc Surg.

[4] Murphy GJ, Angelini GD (2004). Insights into the pathogenesis of vein graft disease: lessons from intravascular ultrasound. Cardiovasc Ultrasound.

[5] Motwani JG, Topol EJ (1998). Aortocoronary saphenous vein graft disease: pathogenesis, predisposition, and prevention. Circulation.

[6] Yang Z (1997). Different effects of thrombin receptor activation on endothelium and smooth muscle cells of human coronary bypass vessels. Implications for venous bypass graft failure. Circulation.

[7] Sopek-Merkaš I (2022). ANTIPLATELET THERAPY AFTER CORONARY ARTERY BYPASS GRAFT SURGERY - UNEVENNESS OF DAILY CLINICAL PRACTICE. Acta Clin Croat.

[8] Valgimigli M (2018). 2017 ESC focused update on dual antiplatelet therapy in coronary artery disease developed in collaboration with EACTS. Eur J Cardiothorac Surg.

[9] Cheung-Flynn J (2017). Limiting Injury during Saphenous Vein Graft Preparation for coronary arterial bypass prevents metabolic decompensation. Sci Rep.

[10] Osgood MJ (2014). Surgical vein graft preparation promotes cellular dysfunction, oxidative stress, and intimal hyperplasia in human saphenous vein. J Vasc Surg.

[11] Weaver H (2012). Oxidative stress and vein graft failure: a focus on NADH oxidase, nitric oxide and eicosanoids. Curr Opin Pharmacol.

[12] de Vries MR (2016). Vein graft failure: from pathophysiology to clinical outcomes. Nat Rev Cardiol.

[13] Gluckman TJ (2011). Effects of aspirin responsiveness and platelet reactivity on early vein graft thrombosis after coronary artery bypass graft surgery. J Am Coll Cardiol.

[14] McEnany MT (1982). The effect of antithrombotic therapy on patency rates of saphenous vein coronary artery bypass grafts. J Thorac Cardiovasc Surg.

[15] Sharma GV et al (1983) *The effect of antiplatelet therapy on saphenous vein coronary artery bypass graft patency*. Circulation, 68(3 Pt 2): p. Ii218-21. 6347428

[16] Lorenz RL (1984). Improved aortocoronary bypass patency by low-dose aspirin (100 mg daily). Effects on platelet aggregation and thromboxane formation. Lancet.

[17] Meister W (1984). Low-dose acetylsalicylic acid (100 mg/day) after aortocoronary bypass surgery: a placebo-controlled trial. Br J Clin Pharmacol.

[18] Gavaghan TP, Gebski V, Baron DW (1991). Immediate postoperative aspirin improves vein graft patency early and late after coronary artery bypass graft surgery. A placebo-controlled, randomized study. Circulation.

[19] Goldman S (1988). Improvement in early saphenous vein graft patency after coronary artery bypass surgery with antiplatelet therapy: results of a Veterans Administration Cooperative Study. Circulation.

[20] Ivert T (2017). Platelet function one and three months after coronary bypass surgery in relation to once or twice daily dosing of acetylsalicylic acid. Thromb Res.

[21] Paikin JS (2017). Once versus twice daily aspirin after coronary bypass surgery: a randomized trial. J Thromb Haemost.

[22] Paikin JS (2015). Multiple daily doses of acetyl-salicylic acid (ASA) overcome reduced platelet response to once-daily ASA after coronary artery bypass graft surgery: a pilot randomized controlled trial. J Thromb Haemost.

[23] Rajah SM (1994). Effects of antiplatelet therapy with indobufen or aspirin-dipyridamole on graft patency one year after coronary artery bypass grafting. J Thorac Cardiovasc Surg.

[24] Fuster V (1993). Aspirin as a therapeutic agent in cardiovascular disease. Special writing Group. Circulation.

[25] Mangano DT (2002). Aspirin and mortality from coronary bypass surgery. N Engl J Med.

[26] Gaudino M (2017). Mechanisms, consequences, and Prevention of Coronary Graft failure. Circulation.

[27] Arazi HC (2010). Impaired anti-platelet effect of aspirin, inflammation and platelet turnover in cardiac surgery☆☆☆. Interact Cardiovasc Thorac Surg.

[28] *A randomised, blinded, trial of clopidogrel versus aspirin in patients at risk of ischaemic events (CAPRIE)*. The Lancet, (1996) 348(9038): p. 1329–1339 10.1016/s0140-6736(96)09457-3 8918275

[29] Bhatt DL (2001). Superiority of clopidogrel versus aspirin in patients with prior cardiac surgery. Circulation.

[30] David JL, Limet R (1999). Antiplatelet activity of clopidogrel in coronary artery bypass graft surgery patients. Thromb Haemost.

[31] Lim E (2004). Clopidogrel did not inhibit platelet function early after coronary bypass surgery: a prospective randomized trial. J Thorac Cardiovasc Surg.

[32] Schunkert H (2019). Randomized trial of ticagrelor vs. aspirin in patients after coronary artery bypass grafting: the TiCAB trial. Eur Heart J.

[33] Kulik A (2022). Ticagrelor versus aspirin and vein graft patency after coronary bypass: a randomized trial. J Card Surg.

[34] Kulik A (2022). Ticagrelor versus aspirin 2 years after coronary bypass: observational analysis from the TARGET trial. J Card Surg.

[35] Gao G (2010). Aspirin plus clopidogrel therapy increases early venous graft patency after coronary artery bypass surgery a single-center, randomized, controlled trial. J Am Coll Cardiol.

[36] Mujanovic E (2009). The effect of combined clopidogrel and aspirin therapy after off-pump coronary surgery: a pilot study. Innovations (Phila).

[37] Mannacio VA (2012). Aspirin plus clopidogrel for optimal platelet inhibition following off-pump coronary artery bypass surgery: results from the CRYSSA (prevention of coronary arteRY bypaSS occlusion after off-pump procedures) randomised study. Heart.

[38] Kayacioglu I (2008). The role of clopidogrel and acetylsalicylic acid in the prevention of early-phase graft occlusion due to reactive thrombocytosis after coronary artery bypass operation. Heart Surg Forum.

[39] Sun JC (2010). Randomized trial of aspirin and clopidogrel versus aspirin alone for the prevention of coronary artery bypass graft occlusion: the preoperative aspirin and postoperative antiplatelets in coronary artery bypass grafting study. Am Heart J.

[40] Kulik A (2010). Aspirin plus clopidogrel versus aspirin alone after coronary artery bypass grafting: the clopidogrel after surgery for coronary artery disease (CASCADE) trial. Circulation.

[41] Rafiq S (2017). Thrombelastographic hypercoagulability and antiplatelet therapy after coronary artery bypass surgery (TEG-CABG trial): a randomized controlled trial. Platelets.

[42] Ebrahimi R (2014). Effect of clopidogrel use post coronary artery bypass surgery on graft patency. Ann Thorac Surg.

[43] Fox KA (2004). Benefits and risks of the combination of clopidogrel and aspirin in patients undergoing surgical revascularization for non-ST-elevation acute coronary syndrome: the Clopidogrel in unstable angina to prevent recurrent ischemic events (CURE) Trial. Circulation.

[44] Qu J (2021). Dual antiplatelet therapy with Clopidogrel and Aspirin Versus Aspirin Monotherapy in patients undergoing coronary artery bypass graft surgery. J Am Heart Assoc.

[45] Ebrahimi R (2018). Comparison of outcomes and costs Associated with aspirin ± Clopidogrel after coronary artery bypass grafting. Am J Cardiol.

[46] Gasparovic H (2014). Impact of dual antiplatelet therapy on outcomes among aspirin-resistant patients following coronary artery bypass grafting. Am J Cardiol.

[47] van Diepen S (2017). Dual antiplatelet therapy Versus Aspirin Monotherapy in Diabetics with Multivessel Disease undergoing CABG: FREEDOM insights. J Am Coll Cardiol.

[48] Saw J (2016). Ticagrelor and aspirin for the prevention of cardiovascular events after coronary artery bypass graft surgery. Heart.

[49] Zhao Q (2018). Effect of Ticagrelor Plus aspirin, Ticagrelor alone, or aspirin alone on Saphenous Vein Graft Patency 1 year after coronary artery bypass grafting: a Randomized Clinical Trial. JAMA.

[50] Willemsen LM (2020). Effect of adding Ticagrelor to Standard Aspirin on Saphenous Vein Graft Patency in patients undergoing coronary artery bypass grafting (POPular CABG): a Randomized, Double-Blind, placebo-controlled trial. Circulation.

[51] Sandner S (2022). Association of Dual Antiplatelet Therapy with Ticagrelor with Vein Graft failure after coronary artery bypass graft surgery: a systematic review and Meta-analysis. JAMA.

[52] von Scheidt M (2020). Ticagrelor-based antiplatelet regimens in patients treated with coronary artery bypass grafting: a meta-analysis of randomized controlled trials. Eur J Cardiothorac Surg.

[53] Danek BA (2020). A Randomized Controlled Trial of Prasugrel for Prevention of Early Saphenous Vein Graft Thrombosis. J Invasive Cardiol.

[54] Gupta S (2020). Antiplatelet therapy and coronary artery bypass grafting: a systematic review and network meta-analysis. Interact Cardiovasc Thorac Surg.

[55] Solo K (2019). Antithrombotic treatment after coronary artery bypass graft surgery: systematic review and network meta-analysis. BMJ.

[56] Held C (2011). Ticagrelor versus clopidogrel in patients with acute coronary syndromes undergoing coronary artery bypass surgery: results from the PLATO (platelet inhibition and patient outcomes) trial. J Am Coll Cardiol.

[57] Xu F (2019). Antiplatelet effects of ticagrelor versus clopidogrel after coronary artery bypass graft surgery: a single-center randomized controlled trial. J Thorac Cardiovasc Surg.

[58] Tang Y (2021). Aspirin plus ticagrelor or clopidogrel on graft patency one year after coronary bypass grafting: a single-center, randomized, controlled trial. J Thorac Dis.

[59] Smith PK (2012). Mortality benefit with prasugrel in the TRITON-TIMI 38 coronary artery bypass grafting cohort: risk-adjusted retrospective data analysis. J Am Coll Cardiol.

[60] Bai C (2022). [A randomized controlled trial of indobufen versus aspirin in the prevention of bridging restenosis after coronary artery bypass grafting]. Zhonghua Xin xue guan bing za zhi.

[61] *Optimal antithrombotic therapy following aortocoronary bypass: a meta-analysis*. Eur J Cardiothorac Surg, (1993) 7(4): p. 169–180 10.1016/1010-7940(93)90155-5 8481254

[62] van der Meer J (1993). Prevention of one-year vein-graft occlusion after aortocoronary- bypass surgery: a comparison of low-dose aspirin, low-dose aspirin plus dipyridamole, and oral anticoagulants. The Lancet.

[63] *The effect of aggressive lowering of Low-Density Lipoprotein Cholesterol Levels and low-dose anticoagulation on obstructive changes in Saphenous-Vein coronary-artery bypass grafts*. N Engl J Med, (1997) 336(3): p. 153–163 10.1056/NEJM199701163360301 8992351

[64] Knatterud GL (2000). Long-Term effects on clinical outcomes of aggressive lowering of Low-Density Lipoprotein Cholesterol Levels and low-dose anticoagulation in the Post Coronary Artery Bypass Graft Trial. Circulation.

[65] Capodanno D (2020). Dual-pathway inhibition for secondary and tertiary antithrombotic prevention in cardiovascular disease. Nat Reviews Cardiol.

[66] Mega JL (2012). Rivaroxaban in patients with a recent acute coronary syndrome. N Engl J Med.

[67] Alexander JH (2011). Apixaban with antiplatelet therapy after acute coronary syndrome. N Engl J Med.

[68] Lamy A (2019). Rivaroxaban, aspirin, or both to prevent early coronary bypass graft occlusion: the COMPASS-CABG study. J Am Coll Cardiol.

[69] Sun JC (2011). Randomized trial of fondaparinux versus heparin to prevent graft failure after coronary artery bypass grafting: the Fonda CABG study. J Thromb Thrombolysis.

[70] Deo SV (2013). Dual anti-platelet therapy after coronary artery bypass grafting: is there any benefit? A systematic review and meta-analysis. J Card Surg.

[71] Nocerino AG, Achenbach S, Taylor AJ (2013). Meta-analysis of effect of single versus dual antiplatelet therapy on early patency of bypass conduits after coronary artery bypass grafting. Am J Cardiol.

[72] Neumann FJ (2019). 2018 ESC/EACTS guidelines on myocardial revascularization. Eur Heart J.

[73] Sousa-Uva M (2018). 2017 EACTS guidelines on perioperative medication in adult cardiac surgery. Eur J Cardiothorac Surg.

[74] Kulik A (2015). Secondary Prevention after coronary artery bypass graft surgery. Circulation.

[75] Malm CJ (2023). Dual or single antiplatelet therapy after coronary surgery for acute coronary syndrome (TACSI trial): Rationale and design of an investigator-initiated, prospective, multinational, registry-based randomized clinical trial. Am Heart J.

[76] Yuan X (2023). Multicentre, randomised, double-blind, parallel controlled trial to investigate timing of platelet inhibition after coronary artery bypass grafting: TOP-CABG trial study. BMJ Open.

